# Protein-Encoding Chemically Modified mRNAs for Musculoskeletal Tissue Regeneration and Repair

**DOI:** 10.3390/jfb17040167

**Published:** 2026-04-01

**Authors:** Britney S. Force, Xueqin Gao, Johnny Huard

**Affiliations:** Linda and Mitch Hart Center for Regenerative and Personalized Medicine, Steadman Philippon Research Institute, Vail, CO 81657, USA; bforce@sprivail.org

**Keywords:** chemically modified protein-encoding mRNA, bone, cartilage, tendon, skeletal muscle, intervertebral disc, biomaterials

## Abstract

Musculoskeletal disorders and injuries are highly prevalent and encompass a broad range of conditions, including bone fractures and segmental defects, tendinopathies and tendon injury, and cartilage disorders such as osteoarthritis, cartilage defects, and intervertebral disc disease. These conditions can arise from diverse causes including trauma and injury, tumor resection, congenital abnormalities, and age-related degeneration. In the past decades, administration of chemically modified mRNA (cmRNA) encoding growth factors and transcriptional regulators has demonstrated effectiveness in repairing musculoskeletal tissues in preclinical studies. This review summarizes recent advancements in bone, tendon, cartilage, intervertebral disc, and muscle regeneration achieved through the localized delivery of protein-encoding mRNAs to express therapeutic target proteins. Delivery of cmRNA encoding growth factors such as BMP-2, BMP-9, VEGF, FGF-18, and IGF-1, or transcriptional regulators including Runx1, to various animal models has shown beneficial effects on bone, tendon, cartilage, and muscle injury repair in preclinical models. Alongside these progresses, the advantages and disadvantages of applying chemically modified mRNA for musculoskeletal tissue regeneration are also discussed. While studies show the promise of cmRNA for therapeutic applications in orthopedic tissue regeneration, more research is required to optimize growth factors and delivery methods, as well as validate long-term safety and efficacy prior to successful translation into new therapies to benefit patients.

## 1. Introduction

Disorders of the musculoskeletal system are a major public health burden and a leading cause of disability, affecting an estimated 27.7% of the population worldwide in 2014 [1]. Regeneration of musculoskeletal tissues including bone, tendon, cartilage, intervertebral disc, and skeletal muscle remains a significant clinical challenge, particularly in terms of large segmental defects, chronic degeneration, or impaired healing. Current treatment strategies such as autografts, allografts, recombinant growth factors, gene therapy, and surgical reconstruction often carry limitations such as donor-site morbidity, graft supply, variable efficacy, or off-target effects [2]. These challenges have driven increasing interest in messenger RNA (mRNA)-based therapeutics, which offer localized expression of growth factors without the integration risks associated with retroviral, lentiviral-mediated gene therapy or the supraphysiological dose required for recombinant protein therapy.

The application of growth factors using gene therapy, ex vivo cell therapy, and biomaterials has proven effective for musculoskeletal tissue regeneration or repair [3,4]; however, its clinical utility has been limited by the challenges associated with recombinant protein delivery and gene therapy approaches. Currently, recombinant human bone morphogenetic proteins-2 (rhBMP2) (Infusion Graft) and -7 (rhBMP7) have been approved by the FDA for therapeutic applications in bone regeneration, but have since been limited due to adverse side effects impeding clinical applications [5]. The supraphysiological dose required to accommodate for a short in vivo half-life increases the cost of treatment and exacerbates adverse effects [6,7]. In attempts to address these limitations, gene therapy approaches employing viral and non-viral vectors for tissue regeneration have demonstrated efficacy, but are constrained by safety concerns of viral vectors and low transfection efficiencies of non-viral vectors [8,9,10,11].

Following the success of COVID-19 mRNA vaccine in controlling the COVID-19 pandemic, therapeutic mRNA has gained significant momentum as a novel strategy for therapeutic target delivery. Numerous preclinical studies have explored the feasibility of protein-encoding chemically modified mRNA for musculoskeletal tissue repair. A previous review article summarized RNA therapies for musculoskeletal conditions and focused primarily on mRNA delivery vehicles [12]. This review article focuses on the applications of protein-encoding mRNA therapy for regenerating bone, cartilage, intervertebral disc, tendon, and skeletal muscle.

In this review, we used following keywords to perform a literature search in PUBMED: “mRNA and bone defect repair”, “mRNA and cartilage repair, mRNA and intervertebral disc repair or regeneration’; “mRNA and tendon repair”; “mRNA and muscle regeneration”. The search results are summarized and categorized based on tissues as following sections.

## 2. Protein-Encoding mRNA for Bone Regeneration

### 2.1. Protein-Encoding mRNA-Based Therapeutic Strategies for Craniomaxillofacial Bone Regeneration

Large osseous defects of the craniomaxillofacial region, often caused by trauma, infection, tumor resection, or skeletal abnormalities, are difficult to regenerate and remain a significant clinical challenge as they can lead to significant physical and psychological impacts [13,14]. Current clinical treatment methods include but are not limited to allografts, autografts, graft-substitutes, or the application of recombinant growth factors such as bone morphogenetic proteins (BMPs) [13]. Limitations to each of these techniques necessitate novel treatment options. mRNA therapy for bone repair has the potential to overcome limitations of existing treatment strategies by enabling the localized expression of BMPs and/or other various angiogenic and osteogenic gene targets.

#### 2.1.1. BMP-Encoding mRNA Delivery for Calvarial Bone Regeneration

Recombinant forms of bone morphogenetic proteins, particularly BMP-2 and BMP-9, have been widely investigated for their applications in bone regeneration [15,16,17,18]. Therefore, chemically modified mRNA encoding BMPs represented some of the earliest mRNA-based attempts for bone regeneration. Among the BMP family, BMP-2 is the most effective BMP [17] and has been widely investigated in mRNA-based bone regeneration.

Elangovan S et al. first investigated the effects of chemically modified ribonucleic acid (cmRNA) encoding BMP-2 (5′ cap modification (ARCA; 7-methyl (3′-O-methyl) GpppGm7G (5′)ppp(5′)G) and triphosphate modifications (25%: 2-thiouridine-5′-triphosphate and 5-methylcytidine-5′-triphosphate (s2U(0.25)m5C(0.25)) and 100%: pseudouridine-5′-triphosphate and 5-methylcytidine-5′-triphosphate (Ψ(1.0)m5C(1.0)) delivered using polyethylenimine (PEI) for inducing osteogenic differentiation of human bone marrow mesenchymal stem cells (hBMMSCs) in vitro, followed by an in vivo evaluation of cmRNA-based therapy for the repair of a rat calvarial bone defect model in comparison with conventional plasmid DNA gene therapy. The polyplex were injected into a collagen scaffold and implanted into a 5 mm diameter calvarial bone defect in rats. In vitro results demonstrated a two-fold increase in BMP-2 protein expression by transfected hBMMSCs compared to PEI-pDNA at the same dose. In vivo, at 4 weeks post implantation, μCT results revealed partial defect healing and increased bone volume to trabecular volume (BV/TV) in defects treated with PEI-cmRNA encoding BMP-2 (25 µg BMP-2 cmRNA) polyplexes compared to 25 µg BMP-2 pDNA polyplexes and significantly increased healing compared to empty defects. Histology analysis further validates extensive bone bridging with more mature mineralized bone tissue in the BMP-2 PEI-cmRNA group, while the pDNA group only showed minimal formation of new bone tissue in the defect margin [19].

Surisaeng T et al. loaded BMP-2 cmRNA (nucleoside modifications: replacement of uridine-5′-triphosphate with m1Ψ-5′-triphosphate) complexed with lipid nanoparticles (LNPs) on either silk fibroin (SF) or gelatin silk fibroin (G) scaffolds and implanted them within a 4 mm calvarial bone defect. Results at 4 weeks post-transplantation showed an increased quantity of mineralized bone matrix within a 5 mm calvarial bone defect, increased percentages of calvarial defect closure, and increased BV/TV compared to empty defects. Using the SF scaffold, only the 15 µg dose of BMP-2 mRNA showed a statistically significant increase in BV/TV, while using the G scaffold all BMP-2 mRNA doses (1.5, 5, and 15 µg) significantly increased BV/TV. However, only partial defect closure was achieved at 15 µg BMP-2 mRNA dosage using either scaffold (57 or 58% closure). This study further demonstrated that implantation of BMP-2 cmRNA-transfected rat bone marrow mesenchymal stem cells (rBMSCs) or rat fibroblasts resulted in near-complete defect healing compared to partial healing of the delivery of BMP-2 mRNA alone [14]. These results demonstrated that the presence of cells that continuously express BMP-2 is very important. Collectively, these studies demonstrated the efficacy of cmRNA encoding BMP-2 in promoting calvarial bone regeneration and its advantages over conventional plasmid DNA therapy.

In addition to BMP-2 mRNA, others have also investigated BMP-9 mRNA for calvarial bone defect repair. Khorsand B et al. compared the regenerative capacity of nanoplexes of polyethylenimine (PEI)-delivered BMP-9 cmRNA (10 µg) (5′ cap modification: ARCA; 7-methyl (3′-O-methyl) GpppGm7G (5′)ppp(5′)G and triphosphate modification: 100% pseudouridine-5′-triphosphate and 5-methylcytidine-5′-triphosphate (Ψ(1.0)m5C(1.0)) and BMP-9 pDNA (10 µg) incorporated into perforated collagen membranes (PCMs) in a critical-sized (5 mm) calvarial defect model in rats for 4 weeks. PEI-cmRNA encoding BMP-9 loaded on PCMs significantly increased mineralized bone and BV/TV relative to the scaffold only group, and a trended increase in BV/TV was observed compared to the PEI-pDNA-PCM treatment group [20]. The same research team compared cmRNA encoding BMP-2 and BMP-9 (cmRNA created using the same modifications as described previously) complexed with PEI at 50 µg dose and injected the PEI-cmRNA into collagen scaffolds that were then freeze dried for subsequent use for in vivo bone regeneration. The freeze-dried collagen scaffolds were applied to 5 mm rat calvarial defects. At four weeks following treatment, it was found that both BMP-2 and BMP-9 cmRNAs regenerated significantly more bone compared to an empty defect (control) with complete defect closing. Although there was no difference in callus formation or BV/TV between BMP-9 and BMP-2 cmRNA groups, the connectivity density was twofold greater, and more bone tissue was discovered in the BMP-9 cmRNA group compared to the BMP-2 cmRNA group [21]. These findings suggest that BMP-9 may be a more effective growth factor than BMP-2 for bone regeneration, when delivered in cmRNA form.

#### 2.1.2. Other Protein-Encoding cmRNA to Promote Angiogenesis and Osteogenesis for Craniofacial Bone Repair

While BMPs have been the most extensively investigated in terms of bone repair, other growth factors including fibroblast growth factor-2 (FGF-2), platelet-derived growth factor-BB (PDGF-BB), transforming growth factor beta-3 (TGFβ-3), runt-related transcription factor 2 (Runx2), and vascular endothelial growth factor (VEGF) are also potential gene targets for chemically modified mRNA therapy.

##### Co-Delivery of BMP mRNA With Additional Growth Factor mRNAs for Craniofacial Bone Defect Repair

Geng Y et al. investigated the synergistic effects of VEGF and BMP-2 cmRNA (full replacement of uridine-5′-triphosphate with N1-methylpseudo-uridine-5′-triphosphate) for bone repair. In vitro assay demonstrated VEGF and BMP-2 secretion by cmRNA lasted for 48 h. Furthermore, BMP-2 mRNA-transfected rat bone marrow stem cells (rBMSCs) (2 µg mRNA-transfected 2 × 10^5^ cells) demonstrated increased osteogenic potential in vitro. In vivo analysis showed enhanced critical-sized (5 mm) defect repair in rat calvarial bones at 4 weeks post-treatment when using a collagen scaffold. VEGF and BMP-2 cmRNA-transfected rBMSCs synergistically enhanced bone defect repair compared to BMP-2 mRNA-transfected rBMSCs by simultaneously promoting osteogenesis and angiogenesis, resulting in improved bone healing and almost complete defect closure. Additionally, the regenerated bone with BMP-2 and VEGF cmRNAs showed higher gene expression of osteogenic-related genes and angiogenesis-related genes (CD31) as demonstrated by immunohistochemistry and Western blot [22].

Similarly, Tsou HK et al. explored the effects of co-application of BMP-2 mRNA (10 µg) and TGFβ-3 mRNA (unknown amount) loaded on polyethylene glycol (PEG) (M.W. = 43,000)-poly-N-[N′-(2-aminoethyl)-2-aminoethyl]aspartamide, which possesses two repeating aminoethylene units (PEG-PAsp(DET)) block copolymer nanomicelles on a gelatin sponge scaffold on the healing of a 4 mm calvarial defect in 8 weeks old male mice. At two weeks post administration, only a small bone island had formed in the BMP-2 + TGFβ-3 mRNA group, while there was no bone formation in the BMP-2 mRNA group. The BMP-2 and TGFβ-3 mRNA combined group regenerated over 10-fold new bone volume (BV) at 8 weeks compared to the BMP-2 mRNA nanomedicine administration alone. However, the defects were only partially healed with bone volume/defect volume ratios around 12% in the combined group [23]. These results indicated a potential synergistic relationship between TGFβ-3 and BMP-2 in enhancing endochondral bone repair, likely due to the role of TGFβ-3 in promoting chondrogenesis.

Surisaeng T et al. evaluated the effect of BMP-2 cmRNA applied alone or in combination with FGF-2 and PDGF-BB cmRNA (uridine-5′-triphosphate replaced with m1Ψ-5′-triphosphate) for calvarial bone defect repair. At 4 weeks post-transplantation, BMP-2 cmRNA significantly enhanced bone regeneration in the 5 mm calvarial defects, while neither FGF-2 nor PDGF-BB cmRNA alone promoted bone repair. Surprisingly, co-delivery of PDGF-BB cmRNA with BMP2 cmRNA suppressed the bone regenerative effects of BMP-2 cmRNA [14]. In contrast, both PDGF-BB and FGF-2 in their recombinant protein forms have previously been shown in enhancing bone regeneration [24,25]. Collectively, these findings indicate that co-delivery of selective growth factors such as VEGF or TGFβ-3 can further enhance bone formation, whereas other factors such as FGF-2 or PDGF-BB may be ineffective or inhibitory.

##### cmRNA Encoding RUNX2 and VEGF for Craniofacial Bone Defect Repair

In addition to the co-delivery of mRNA encoding growth factors with BMP-2, combinations of other mRNAs encoding growth factors such as Runx2 and VEGF have also been investigated for promoting osteogenic differentiation and craniofacial bone defect repair. Zhang M et al. injected polyethylene glycol (PEG) and polyamino acid (Poly(N-[N′-(2-aminoethyl)-2-aminoethyl]aspartamide)) (PEG-PAsp(DET)) nanomicelles loaded with Runx2 (10 µg) and VEGF mRNAs (10 µg) to a 4 mm mandibular defect model. mRNA administration began at 7 days after the creation of the defect, continuing once weekly, with µCT imaging, for four weeks post-surgery. Results revealed that while each mRNA individually significantly enhanced bone regeneration, their combined administration produced a synergistic effect, further promoting bone formation compared with either Runx2 or VEGF mRNA alone, as evidenced by increased BV and BV/TV in µCT and histology analyses. However, complete defect healing was not achieved, even in the combined RUNX2 and VEGF mRNA treatment groups [26].

##### Natural mRNA Delivered with Exosomes for Calvarial Bone Defect Repair

Yang Z et al. designed BMP-2 mRNA-enriched engineered exosomes loaded into hydrogel to achieve sustained release for more efficient and safe bone regeneration. BMP-2 mRNA was enriched into exosomes by selective inhibition of translation in donor cells, in which NoBody (non-annotated P-body dissociating polypeptide, a protein that inhibits mRNA translation) and modified engineered rat BMP-2 plasmids were co-transfected into 293T cells. Exosomes were then isolated from co-transfected cells and named ExoBMP2 + NoBody. In vitro experiments confirmed that ExoBMP2 + NoBody had higher abundance of BMP-2 mRNA and thus stronger osteogenic induction capacity. When loaded into GelMA hydrogel via ally-L-glycine modified CP05 linker, the exosomes were sustained, released, and demonstrated prolonged expression of BMP-2 when endocytosed by the recipient cells. In vivo, ExoBMP2 + NoBody-loaded GelMA applied to a 5 mm rat critical-sized calvarial defect model displayed greater capacity in promoting bone regeneration than the control group after 4- and 8- weeks post-implantation [27]. This approach takes advantage of natural BMP-2 mRNA and showed no risk of incurring immune response.

### 2.2. Protein-Encoding MRNA Therapy for the Repair of Long Bone Defects

#### 2.2.1. Protein-Encoding CmRNA for Repair of Non-Critical-Sized Long Bone Defects or Fractures

The physiological mechanism of fracture healing is a complex, innate, regenerative process. However, fracture, if not treated properly, can potentially result in delayed or non-union fractures and even cause mortality in elderly individuals [28]. Non-union is estimated to occur in 2% of all fractures, with instances of non-union rising to as high as 20% for certain long bone shaft fracture injuries [29]. Several mRNA therapy studies on femoral or tibial fractures in animal models have aimed to address this clinical need.

Balamayor E et al. first reported that BMP-2 cmRNA expressing stem cells demonstrated enhanced osteogenic differentiation in vitro. BMP-2 cmRNA (2.5 µg) (modifications: 25% of cytidine-5′-triphosphate and uridine-5′- triphosphate were replaced by 5-methylcytidine-5′-triphosphate and 2-thiouridine-5′-triphosphate) delivered via Fibrin gel + C12-EPE/hBMP-2 cmRNA lipoids was applied to a 3 mm non-critical-sized rat femoral defect model, resulting in the increased formation of mineralized callus volume and regenerated bone as early as 2 weeks at a very low dose compared to the fibrin-only control defect. Additionally, more mature bone was observed in the BMP-2 cmRNA group as evidenced by the formation of haversian canals and the presence of bone lining cells in the bone tissue [30]. Badieyan ZS et al. further reported that vacuum-dried cmRNA-loaded collagen sponges, termed transcript activated matrices (TAMs), exerted 100% transfection efficiency of reporter target protein and can translate target protein for 6 days. TAMs loaded with 2.5 µg BMP-2 cmRNA (chemically modified ribonucleotides 5-methyl-CTP and 2-thio UTP) applied to a 2 mm drill hole non-critical rat femur defect resulted in a statistically significant increase in bone callus volume, bone area, and osteoid volume as confirmed by μCT and histomorphometric analysis at two weeks post-implantation [31]. These findings indicate the therapeutic potential of BMP-2-encoding mRNA in non-critical size long bone defect repair.

In addition to BMP-2 cmRNA, other osteogenic factors for mRNA therapy have also been explored in tibia fracture healing. Nelson AL et al. examined the activation of the Wnt/β-catenin pathway using β-catenin^GOF^ cmRNA (modified nucleoside: uridine replaced with N1-methyl-pseudouridine) encapsulated within SM-102 lipid nanoparticles to promote endochondral ossification during fracture healing in mice. After injection of β-catenin^GOF^ mRNA/LNP at 6 days post-fracture at various doses (10 to 45 µg in 25–30 µL volume) to a mouse tibia fracture site, results recorded at 2 weeks following fracture and stabilization showed that 45 µg β-catenin^GOF^ cmRNA promoted bone fracture healing as revealed by increased bone formation and decreased cartilage composition and a higher BV/TV compared to the control group. Similar effects were observed with 25 ng of rWnt3A delivered at the fracture site [32].

#### 2.2.2. Protein-Encoding mRNA for Repair of Critical Sized Segmental Long Bone Defects

Critical-sized segmental bone defects remain a significant clinical challenge in terms of treatment. These defects, which can be caused by tumor resection, trauma, infection, or other diseases, currently rely on surgical treatment such as bone autografts, allografts, or free flaps [26,33]. The delivery of cmRNA has shown therapeutic potential in animal models to repair critical size segmental bone defects of the femur.

De La Vega RE et al. evaluated BMP-2 cmRNA (35% of uridine residues were replaced with 5-iodo-uridine and 7.5% of cytidine residues were replaced with 5-iodo-cytidine) delivery versus recombinant BMP-2 protein (rhBMP-2) in a rat critical sized femoral defect model. Various doses of BMP-2 cmRNA or rhBMP-2 protein were loaded on a collagen sponge and implanted into a 5 mm stabilized defect covered with a deep muscle pouch. As early as 4 weeks post-surgery, BMP-2 cmRNA promoted dose-dependent bone regeneration, with complete defect bridging observed at 50 μg but not at lower doses. The 50 μg cmRNA group exhibited greater BV/TV and Tb.N at 8 weeks after surgery, faster mechanical recovery, initiated bone remodeling faster than control group, and produced superior bone quality when compared with all other groups. Similar results were observed with the application of 11 µg rhBMP-2. Additionally BMP-2 delivered via cmRNA remained localized at the defect with no off-target detection in other tissues or organs and no adverse effects, whereas an ectopic callus was formed in the rhBMP-2 group at 11 µg dosage [34].

In addition to cmRNA applications for long bone defects, the use of unmodified natural BMP-2 mRNA delivered with extracellular vesicles has also been reported. Ma Y et al. used therapeutic small extracellular vesicles (t-sEVs) endogenously loaded with a cocktail of human vascular endothelial growth factor A (VEGF-A) and human bone morphogenetic protein 2 (BMP-2) mRNAs within a customized injectable PEGylated poly (glycerol sebacate) acrylate (PEGS-A) hydrogel for bone regeneration in a rat distal femur critical-size (3 mm × 5 mm) defect model. Using a nanoelectroporation system based on a commercially available track-etched membrane (TM-nanoEP), plasmid DNAs were delivered to human adipose-derived mesenchymal stem cells (hAdMSCs), and subsequently t-sEVs were isolated. It was found that upregulated microRNAs associated with the therapeutic mRNAs (BMP-2 and VEGF-A) are enriched in t-sEVs for enhanced angiogenic-osteogenic regeneration. Results collected at 4 and 8 weeks post-surgery demonstrated localized and controlled release of t-sEVs within the PEGS-A hydrogel led to the retention of therapeutics in the defect site for highly efficient bone regeneration with minimal/low accumulation in other organs [35].

## 3. Protein-Encoding cmRNA for the Regeneration of Articular Cartilage

### 3.1. Protein-Encoding cmRNA for Post-Traumatic Osteoarthritis Repair

Osteoarthritis (OA) is an increasingly prevalent cause of disability in older adults [36,37]. While conventional, non-surgical treatment options are aimed at symptom management, approaches that are capable of regenerating or slowing the degeneration of cartilage remain limited and are still under active research [38]. In the context of mRNA-based approaches for chondrogenesis, several mRNAs encoding proteins such as runt-related transcription factor-1 (Runx1), insulin-like growth factor-1 (IGF-1), fibroblast growth factor-18 (FGF-18), and transforming growth factor beta-1 (TGFβ-1) have been investigated.

Runx1 represents a promising mRNA therapeutic target as it has a regulatory role in articular cartilage maintenance and OA development [39,40]. Overexpression of Runx1 within the knee joint of an OA mouse model has been shown to have a protective effect on articular cartilage [41]. Aini L et al. first reported using mRNA encoding Runx1 for cartilage repair. Two polyethylene glycol (PEG)-polyamino acid block copolymer-based polyplex nanomicelles were developed: PEG-PAsp (DET) and PEG-PAsp(TET). PEG-PAsp (DET) promoted strong reporter gene (Luciferase) expression at 24 h, whereas PEG-PAsp(TET) induced target reporter gene expression at 4 h that persisted for up to 96 h. Intra-articular injection of Runx1 mRNA (1 µg in 20 µL volume every 3 days for one month) via PAsp(DET) nanomicelles improved MCL- and MM-transection-induced OA, but the effects did not reach statistical significance compared to GFP mRNA. However, administration of an equivalent dose of Runx1 mRNA using PEG-PAsp(TET) nanomicelles in the same mouse OA model induced target protein expression in superficial and middle zones of the articular cartilage, significantly suppressed OA development (decreased OARSI histology score) and formation of osteophytes, and increased SOX9 and COL2 expression and cell proliferation [42]. Subsequently, Pezzotti G et al. used the same animal model to deliver Runx1 mRNA using PAsp(DET) nanomicelles and evaluated the outcomes using Raman spectroscopic analysis, which revealed cartilage regeneration at the molecular level at 2- and 4- weeks post-surgery. The mechanism of cartilage restoration was attributed to activation of remaining chondrocytes by Runx1 leading to increased hyaluronic acid synthesis and restoration of organized collagen secondary structures [43].

Treatment with IGF-1 cmRNA has also shown promising results. The intra-articular injection of IGF-1 cmRNA (modification: uridine fully replaced by N1-methylpseudouridine)-transfected adipose derived stem cells (ADSCs) (2 × 10^5^) at 1 and 2 weeks post-surgical destabilization of medial meniscus (DMM) prevented the degeneration of articular cartilage as proved by decreased OARSI histology score and increased COL2 and ACAN expression [44].

FGF-18 mediates proliferation and differentiation of chondrocytes and has been investigated in its recombinant form (rhFGF-18) in clinical trials for the regenerative treatment of OA [45].

Huang K et al. designed a new proprietary lipid nanoparticle TG6A with branched tails and five ester bonds. This TG6A LNP significantly increased reporter GFP mRNA expression in transfected MSCs by 9- and 41-fold compared to commercialized DLin-MC3-DMA and ALC-0315 lipid nanoparticles, respectively. MSCs transfected with TG6A LNP-encapsulated circular FGF-18 mRNA exhibited enhanced chondrogenic differentiation in an in vitro 3D pellet culture differentiation model. In a rat anterior cruciate ligament (ACL) transection-induced OA model, transplantation with circular FGF18-engineered MSCs (2.5 mg/mL in 100 µL HA) at 6 weeks post-surgery not only protected cartilage from damage, but also improved repair as evidenced by thicker cartilage layers, reduced histopathological scores, maintenance of zone structure, and higher type II collagen and extracellular matrix (ECM) deposition compared to the untreated control [46].

Furthermore, Sun M et al. developed a novel articular cavity-localized lipid nanoparticle (LNP) named WG-PL14. This optimized formulation has a nearly 30-fold increase in mRNA expression as well as better articular cavity enrichment compared to commercial MC3 lipids after intra-articular injection. Treatment of anterior cruciate ligament transection-induced (ACLT) OA mice with an intra-articular injection of 2 µg (in 20 µL/knee, once a week for 3 weeks) cmRNA encoding rhFGF-18 (unspecified modifications by mRNAid to improve stability and immunogenecity) complexed with WG-PL14 LNPs reduced OA progression. Delivery of rhFGF-18-encoding mRNA to the joint reduced osteophyte formation and increased tibial subchondral bone BV/TV, trabecular thickness (Tb.Th) and bone mineral density [47]. Consequently, rhFGF-18 mRNA delivered on optimized LNPs also improved pain response, upregulated cartilage extracellular matrix (ECM)-related genes such as COL2 and ACAN, and decreased COL1, MMP13 and IL1β [47]. More recently, Kong K et al. reported that LNP/FGF18 mRNA can deliver FGF-18 cmRNA (uridine replaced by N1-methyl pseudouridine (m^1^ψ)) deeper within cartilage than proteins. Intra-articular injection of FGF18 mRNA/LNP (6 µg weekly in 20 uL) or 500 ng FGF18 protein (in 20 µL) for 8 weeks in the joint of DMM-induced age-related OA models improved OA symptoms via activation of the FOXO3a-autophagy pathway, protecting chondrocytes from degeneration and senescence [37].

Together, these findings highlight the potential of FGF-18 mRNA, delivered directly or via engineered MSCS, for the treatment of OA.

### 3.2. Protein-Encoding cmRNA for Osteochondral Defect Repair

In addition to using cmRNA for traumatic osteoarthritis treatment, cmRNA has also been tested in an osteochondral defect model.

TGFβ-1 mRNA plays an important role in cartilage homeostasis and has been investigated for use in cartilage defect repair [48]. Fontana G et al. developed mRNA delivery vehicles using mineral coated microparticle (MCM) and fluoride MCM (FMCM) and mimicked a clinical setting using bone marrow aspirate concentrate (BMAC) as an ex vivo mRNA carrier. After complexing TGFβ-1 mRNA (at an unspecified dose) with FMCMs, the mRNA was used to transfect BMAC. The transfected BMAC was then combined with autologous peripheral blood to form a clot, which was implanted into a 2.7 mm condyle osteochondral defect created in a rabbit knee. Rabbits treated with the BMAC-TGFβ-1 mRNA regenerated cartilage with increased type II collagen and glycosaminoglycan deposition, as well as reduced COL1 formation; however, no improvement in Odriscoll’s histology scores was observed [49]. Collectively, these studies demonstrate that mRNA-based delivery of chondrogenic factors represents a promising approach to the protection and restoration of osteoarthritic cartilage and osteochondral defect repair.

## 4. Protein-Encoding mRNA for Intervertebral Disc Regeneration

Intervertebral disc degenerative disease (IVDD) affects millions of people worldwide and is a major factor contributing to low back and neck pain and associated disability. Current treatments mainly focus on symptom management using surgical approaches, yet no treatments address disc regeneration, as the underlying mechanisms leading to IVDD are still elusive.

Lin C et al. used PAsp(DET) to form nanomicelles complexed with Runx1-mRNA, which were then injected (4 µg in 6 µL) into a needle-puncture-induced disc injury model in the rat coccygeal 4–5 disc. They found that injection of nanomicelle-Runx1 mRNA complexes reduced inflammation and increased intradiscal Runx1 expression compared to naked Runx1 mRNA injection, which in turn reduced disc height loss, prevented fibrous tissue formation, increased disc hydration content as revealed by MRI, and enhanced nucleus pulposus ECM production of COL2 and ACAN at 2 and 4 weeks post-injury [50,51]. Antony J S et al. designed a new IGF-1 mRNA construct using neurotrophin-3 (NTF3) signal peptide, pro-human IGF-1 domain, and a full-length IGF-1 mRNA coding sequence (Cpd.3) to enhance IGF-1 secretion. This IGF-1 mRNA construct secreted higher levels of mature IGF-1 protein than the IGF-1 natural signal peptide sequence (Cpd.1) following transfection of multiple cell lines. Starting at one month after the induction of a lumbar spine disc degeneration model using needle puncture and disc denucleation (disc herniation model), injections of 20 µg of Cpd.3 IGF-1 mRNA in 20 µL were performed on days 30, 37, 44 and 51 using a citrated saline buffer. At 90 days post-surgery, the disc height index and histology scores were significantly higher than saline-injected discs, although still inferior to uninjured controls [52].The success of these studies using mRNA therapy in ameliorating IVDD progression demonstrated potential therapeutic benefits of delivery of Runx1 and IGF-1 mRNAs.

## 5. Protein-Encoding mRNA for Tendon Regeneration and Healing

Tendon-related injuries account for 30% of musculoskeletal injury-related conditions [53], yet advancements in repair techniques are necessary as surgical interventions have high rates of re-injury and attempts to reduce inflammation have variable success [53]. The use of novel therapeutics such as cmRNA presents a potential innovative approach to the healing of tendon injuries. Several studies thus far have explored the potential to achieve localized protein expression through cmRNA transfection in tendon tissues.

### 5.1. Protein-Encoding mRNA for Tendon Defect Repair

Groth K et al. demonstrated that injection of naked cmRNA encoding the reporter gene Luciferase in saline solution into tendon tissue explants in a variety of species including ovine, equine, bovine, rats, and porcine resulted in high expression of reporter gene (BLI) in the injection site. The authors further observed dose-dependent expression of reporter genes following injection in saline. They also proved the expression of Laz reporter gene and human BMP-7 protein in the explant tissues. They subsequently tested different solvents for injection of 50 µg Luc cmRNA in bovine explants and found HEPES-buffered glucose (HBG 5%) reached maximum expression after 24 h among all solvents tested. Furthermore, 5% glucose in HEPES or saline reached equal Luciferase expression levels while DreamfectGold and Branched PEI did not induce Luciferase expression compared to an untreated tendon. The authors further tested the injection of naked cmRNA in the tendon of rats in vivo and found the expression of the reporter gene Luciferase (Luc) peaked in 24 h and declined to baseline at 7 days. Finally, human BMP-7 cmRNA (100 µg, unspecified modifications) injected into a ruptured rat calcaneal tendon resulted in significantly high expression of hBMP-7 at 2 days and decreased collagen III expression in the regenerating tissues at day 7 [54]. Moreover, Herbst E et al. performed a pilot study by using 5% HEPES-buffered glucose as injection solution to inject 110 µg (1 mg/mL cmRNA in 110 µL, modifications: 25% 2-thio-UTP and 25% 5-methyl-CTP) of basic fibroblast growth factor (bFGF) cmRNA or Luciferase cmRNA into a proximal and distal 2 mm Achilles tendon defect in rats. The results showed Luciferase expression can be detected for up to 3 days post-treatment. The bFGF cmRNA group demonstrated increased tendon stiffness at 14 days compared with the Luciferase cmRNA control group, reaching levels similar to that of a contralateral healthy side tendon. No differences were observed in terms of load to failure or in the expression of COL1,2,3,4 or procollagen I compared with the Luciferase cmRNA-treated group. The bFGF cmRNA-treated group exhibited more oval-shaped nuclei than the Luciferase cmRNA control group. No side effects were detected [55]. It is noteworthy to mention that these earlier studies injected naked mRNA without any lipid nanoparticles or other delivery carriers, thereby requiring large doses of cmRNA.

Sturm L et al. optimized the efficiency of a cationic, hyperbranched poly(amidoamine)-based nanoparticles to deliver tdTomato cmRNA (unspecified modifications) to various cells including rat tendon-derived stem/progenitor cells (rTDSPCs). It was found that nanoparticle (NP)-mediated mRNA delivery to tendon-derived cells was effective in a dose-dependent manner. The transfection efficiency for rTDSPC was 18.13% ± 12.07. Moderate amounts of NPs enhanced transfection efficiency, while higher doses caused cytotoxicity, evidencing the need to balance effective delivery with cell viability. Furthermore, an increase in the mRNA loading ratio (2:50, 4:50, or 6:50 *w*/*w* mRNA:NPs) had no impact on transfection efficiency [56].

More recently, Faustini B et al. aimed to improve tendon repair by targeting two pathways using injectable poly(amidoamine)-based polymers (ps-PAAQ) as nanoparticle (NP) carriers to deliver chemically modified ARCA-capped mRNAs (cmRNAs) (35% of uridine residues were substituted with 5-iodo-uridine and 7.5% of cytidine residues were replaced by 5-iodo-cytidine) encoding Interleukin-1 receptor antagonist (IL1RA) and Platelet-Derived Growth Factor-BB (PDGF-BB). In vitro study demonstrated that delivery of 0.5 µg to 1 µg of target cmRNA using the aforementioned nanoparticles efficiently induced target protein expression in tendon cells and explant tendon tissues. PDGF-BB cmRNA-transfected cells exhibited enhanced proliferation and migration, as well as increased proliferation within tendon explants. IL1RA cmRNA-transfected cells exhibited reduced levels of pro-inflammatory cytokines. In vivo, the authors found that delivery of 1.9 µg of target cmRNA at 7 days post-surgery resulted in the most effective target protein expression and patellar tendon defect repair compared to delivery immediately after surgery or a staged approach involving immediate delivery of IL1RA cmRNA followed by PDGF-BB cmRNA at 7 days in a pilot study. Furthermore, simultaneous delivery of 1.9 µg PDGF-BB cmRNA and 1.9 µg IL1RA cmRNA using Ps-PAAQ nanoparticles at 7 days post-surgery improved tendon repair in a rat patellar tendon window defect model (2 mm diameter). The beneficial effects were mediated by a reduction in inflammatory cells (CD68), decreased expression of pro-inflammatory cytokine (COX2) at early time points post-injury, reduced levels of fibrotic markers (S100a4), and an enhancement in repair tissue structure [57].

### 5.2. Protein-Encoding mRNA for Tendinopathy Regeneration

To more closely resemble the clinical scenario of acute tendinopathy in sheep, Groth K et al. injected collagenase 1 diluted in a fibrin and thrombin solution into superficial digital flexor tendons (SDFT) in sheep to induce tendinopathy. At 6 days post-surgery, cmRNA^Luc^ (at dose of 300, 400, or 500 µg) was injected using 5% HBG injection solution. At 7 days post-surgery, it was found that target gene Luciferase expression did not increase over time in intact tendon, while dose-dependent Luciferase expression was found in the injured tendons [54]. This study indicates injection of cmRNA in the injured tendon can effectively induce target gene expression.

Zhang Y et al. demonstrated that in vitro delivery of mouse IL1RA mRNA to tendon stem cells induced IL1RA protein expression for up to 72 h; suppressed TNFα, IL6, and iNOS expression; and restored Collagen I/III balance as well as cell migration. In vivo, a single injection of 2 µg IL1RA mRNA encapsuled within SM102 lipid nanoparticles (LNPs) in a collagenase I-induced tendinopathy model in mice reduced inflammatory markers and MMP1/13 expression and improved collagen alignment at 1-week post-administration. At 4 weeks post-administration, histology results showed improved collagen organization, increased COL1, decreased COL3, and decreased the COLIII/I ratio compared to the untreated group, and functional recovery was evidenced by the re-establishment of typical gait pattern as normal uninjured mice [58].

Collectively, these studies demonstrated that delivery of cmRNA encoding IL1RA, by targeting inflammation, promotes the regeneration of tendon defects and offers a potential approach for the treatment of tendinopathy. More recent studies have demonstrated that delivering cmRNA via lipid nanoparticles could more effectively induce target therapeutic protein expression with much lower dose of mRNA.

## 6. Protein-Encoding mRNA for Skeletal Muscle Injury Repair

Injuries to skeletal muscle, such as muscle strains, are common in daily life, exercise, and sports. Patients that sustain mild skeletal muscle injuries often achieve full functional recovery, while those with more severe injuries can have prolonged symptoms such as muscle weakness/loss of function that are resistant to traditional treatments [59]. Furthermore, volumetric muscle loss due to traumas such as blast injuries in combative soldiers have no effective treatment [60,61]. Therefore, the application of protein-encoding mRNA for muscle healing and regeneration also has been reported.

Antony J S et al. designed a new IGF-1 mRNA construct using brain-derived growth factor (BDNF) signal peptide, pro-domain of human IGF-1, and a full length IGF-1 mRNA coding sequence (Cpd.2) to enhance IGF-1 secretion. This modified IGF-1 mRNA construct secreted 3.1- to 6.1-fold higher levels of mature IGF-1 protein than IGF-1 natural signal peptide sequence (Cpd.1) after transfection of multiple cell lines. The concentration dependent EC50 was equivalent to clinically approved recombinant protein INCRELEX [52]. Injection of Cpd.2 IGF-1 mRNA (1 µg) to a notexin-induced myotoxic mouse muscle injury achieved functional muscle recovery equivalent to that of 10 µg Cpd.1 IGF-1-mRNA. A dose-dependent injection revealed Cpd.2 IGF-1 mRNA achieved peak effects at a 3 µg dose (bell-shaped effect), whereas Cpd.1 IGF-1 mRNA did not reach a bell-shaped effect even at a 30 µg dose. The healing potency of Cpd.2 IGF-1 mRNA was found to be 15-fold higher than Cpd.1 IGF-1 mRNA in a skeletal muscle injury model [52]. Furthermore, a single injection of Cpd.2 IGF-1 mRNA in a rat punch TA muscle injury model produced IGF-1 protein levels of 18 pg/mg protein within the 4 mm injection site, reaching the therapeutic range and activating the down-stream signaling pathway (pAKT), with effects spreading up to 4–8 mm from the injection site. Human IGF-1 expression was sustained for 3 days, with an approximate 20 h half-life, and activated the down-stream IGF-IR signaling pathway. Furthermore, injection of 3 µg Cpd.2 IGF-1 mRNA significantly increased the expression of early myogenic differentiation markers PAX7 and MYH3, as well as MyoD1, MYH5, and MyoG expression within 72 h in the 4–8 mm from injection site. The late myogenic markers MYH8 and MYH4 did not change within 72 h. This supports the therapeutic effects of Cpd.2 IGF-1 mRNA in this more severe muscle injury model [52].

**Figure 1:**
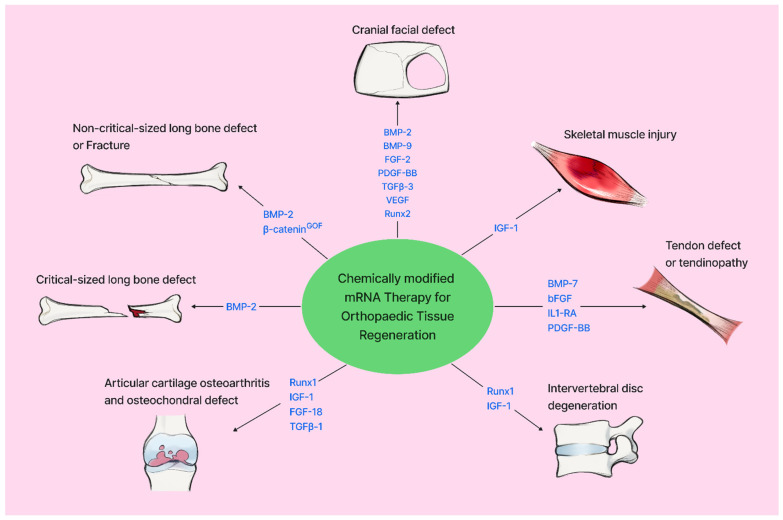
Schematic summary of protein-encoding mRNA for musculo-skeletal repair and regeneration.

Taken together, considerable progress has been made in using protein-encoding cmRNA for musculo-skeletal repair; we have summarized the current progress of using cmRNA for musculoskeletal repair in Table 1. We also summarized the key findings in Figure 1.

## 7. Advantages and Disadvantages of Protein-Encoding mRNA Therapy for Musculoskeletal Tissue Repair

Following the successful use of the COVID-19 mRNA vaccine in the control of the pandemic and the subsequent Nobel Prize recognition for the development of modified mRNA technology, protein-encoding cmRNA has made significant progress in musculo-skeletal tissue repair in the past decade. There has been more advancement in the use of cmRNAs encoding BMPs for bone regeneration than in other musculoskeletal tissues, likely due to the well-confirmed effects of BMP-2 in promoting bone regeneration. BMP-2-encoding mRNA is widely used by scholars and has demonstrated similar efficacy to rhBMP-2 with reduced side effects such as heterotopic ossification formation due to supraphysiological protein doses [34]. The application of cmRNA for articular cartilage repair is also advantageous in that lower doses are required and can be administered via multiple intra-articular injections. This is especially suitable for OA treatment and intervertebral disc regeneration. Furthermore, cmRNA for tendon repair has demonstrated advantages, as it can be injected directly into the tendon injury site, enabling proteins encoded by cmRNA to be expressed in surviving tendon cells and promote tendon repair through paracrine signaling. Protein-encoding cmRNA may also be advantageous for treatment of muscle injuries, especially volumetric muscle loss often accompanied with bone and tendon loss. Multiple cmRNAs encoding proteins of different functions, such as BMP-2 and IGF-1, utilize muscle as a protein-producing organ (similar to the mechanism of the COVID-19 vaccines) and thereby promote the repair of multiple tissues.

However, the application of mRNA therapeutics for musculoskeletal tissue regeneration is not without shortcomings. One limitation is that most of the studies using mRNA therapeutics to repair bone presented better BV/TV and very few presented the total regenerated bone volume. In terms of bone tissue engineering or regeneration, the regenerated bone volume is of higher importance, meaning that complete defect healing is more important than partial healing with a high BV/TV. For the treatment of bone defects, most studies use substantially higher doses of mRNA compared with protein. For example, in treating a rat segmental bone defect model, 50 µg BMP-2 mRNA was required to achieve complete healing, compared to 11 µg BMP-2 protein, with the additional requirement to cover the mRNA/LNP complexes using host muscle tissues [34]. In a mouse tibia fracture model, 45 µg β-catenin mRNA was used and produced effects comparable to using 25 ng Wnt3A protein [32]. The requirement for such high mRNA doses may be due to the lack of host cells within the bone defect after injury, resulting in a limitation of host cell transfection and subsequent degradation of large amounts of unincorporated mRNA. In contrast, protein administration with sustained released biomaterials only required a very small dose of BMP-2 (as little as 1 µg) to achieve complete bridging of a critical-size femoral defect [62] or calvarial bone defect (2ug) [17]. Secondly, once mRNA is applied in the defect, it is uncertain which cells it will transfect. For example, if mRNA/LNP transfects inflammatory cells, they will not undergo osteogenesis. It is also hard to determine the exact quantity of the target protein produced. In contrast, protein delivery can elicit immediate functional effects and even recruit host reparative cells to the defect area. Most studies that track target protein expression use reporter genes, not therapeutic genes. On the other hand, mRNA-transfected stem cells (ex vivo therapy) appeared more effective, likely due to the delivery cells themselves playing an important role in the regenerative process. In this aspect, compared to retroviral or lentiviral vector ex vivo gene therapy, mRNA-transfected cells do not offer extended expression of target protein, but rather transit expression only (maximum 6 days in vitro or in vivo).

Moreover, mRNA therapy appears to be more effective in articular cartilage, intervertebral disc, tendon, and skeletal muscle repair than in bone defect repair, often requiring lower doses and, in some cases, not necessitating lipid nanoparticle delivery systems. This is likely because injury sites in articular cartilage, intervertebral discs, tendons, and skeletal muscle have remaining target cells that can be transfected by mRNA, resulting in therapeutic target protein expression in the injury site and thereby promoting tissue repair more efficiently.

## 8. Perspective

The application of mRNA therapeutics in musculoskeletal regeneration represents a promising and rapidly evolving field. Despite much progress in the applications of protein-encoding mRNA for musculoskeletal tissue regeneration, further optimizations of cmRNAs, delivery methods, and tissue specific protein targets are required prior to clinical translation.

One major barrier to therapeutic use of mRNAs has been the innate immune response incurred by exogenic mRNAs. Nucleoside modifications, such as those used in the development of the COVID-19 vaccine, are intended to prevent reduced protein translation and immunogenicity caused by activation of immune pathways. The modifications that have been investigated in musculoskeletal tissues thus far include U→s2U (25%), C→m5C (25%), U→Ψ (100%), C→m5C (100%), U→m1Ψ (100%) and U→5IU (35%) C→51C (7.5%) with U→m1Ψ (100%) being the most common. While one study compares the use of two substitution combinations (U→s2U (25%), C→m5C (25%) and U→Ψ (100%), C→m5C (100%)) [19], no further direct comparisons have been made to determine the most effective modifications for musculoskeletal tissues specifically. Furthermore, one study explores the use of untranslated region (UTR) alterations to improve translation efficiency [52], showing that further optimizations of both nucleoside substitutions within functional proteins and UTRs are necessitated prior to clinical translation.

Similarly, effective mRNA delivery for musculoskeletal tissues is heavily reliant on the use of delivery modalities/platforms to avoid degradation, immune activation, and sustained delivery. Several types of modalities have been tested in musculoskeletal tissues, however, no direct comparative studies determining the ideal modalities for various tissue types and clinical translation have been reported. As evidenced by the COVID-19 vaccines, the use of such delivery modalities can lead to logistical issues with manufacturing and distribution at the clinical scale. For example, LNP formulations often need cold-chain storage or ultra-low temperatures [63] to maintain stability. While some techniques to maintain shelf stability such as vacuum dried scaffolds [31] have been shown effective in pre-clinical models, mRNA applications for cartilage, intervertebral discs, tendons, and muscles, are primarily administered as liquid-state injectables prompting investigation of other techniques such as lypophilization [64] to improve LNP stability.

Additionally, cmRNA vaccines are advantageous compared to cmRNA for musculoskeletal regeneration, as cmRNA vaccines leverage muscle as a protein-producing organ to release the target antigen into the blood stream, thereby stimulating the immune system to produce cellular and humoral immunity. The immune system does not require large amounts of the target protein or sustained protein release to be effective. On the contrary, long-term expression and retention at the defect site is critical for cmRNA-mediated regeneration of musculoskeletal tissues, emphasizing the need for materials such as scaffolds and hydrogels for sustained release in combination with delivery platforms to protect mRNA from degradation. While the use of scaffolds and hydrogels should be further explored, emerging techniques such as stimuli-responsive release of LNP/cmRNA [65,66] and self-amplifying RNA [67,68] that does not require LNP delivery and can be stored at room temperature should also be investigated for musculoskeletal muscle tissue regeneration.

Furthermore, tissue specific growth factor targets for mRNA therapy in articular cartilage, muscle, and ligaments also warrant further investigation. For example, BMPs have been shown effective in articular cartilage repair using biomaterial mediated-delivery [4,69,70,71,72], but have not been studied using cmRNA delivery for articular cartilage repair. Furthermore, even fewer studies have investigated cmRNA therapy for muscle injury repair. No study has been conducted for meniscus or ligament repair likely due to difficulties in creating injury models and the delivery of target mRNA. Additionally, an under-utilized advantage of therapeutic cmRNA is that it allows for the use of combinatorial growth factor therapies. The synergistic and inhibitory relationships observed between BMP-2 and various growth factors [14,22,23] warrant further investigation of additional combinations to optimize therapeutic efficacy. The contradictory results might be related to the delivery nanoparticle and timing of administration rather than the growth factor’s role in bone defect healing.

Finally, while the short-term safety and efficacy have been evaluated in pre-clinical models, additional research is needed to assess long-term effects, off-target expression, immune responses, and challenges before translation to the treatment of human patients. Whether the modified nucleotides can be uptaken by host cells incorporated into host cells mRNA is also a concern. Future studies should focus on optimizing cmRNA constructs and delivery systems to extend the target protein expression in the injury site, or design cell-specific targeting cmRNA to maximize efficiency and minimize off-target effects and cytotoxicity. Investigations into synergistic effects of growth factor combinations, particularly in tendon and cartilage repair, are warranted and may further enhance regenerative outcomes. Additionally, long-term preclinical and translational studies are needed to address safety, efficacy, and potential clinical applications.

## 9. Conclusions

In summary, protein-encoding mRNA-based therapeutics offer a novel approach to musculoskeletal tissue regeneration that can overcome some limitations of protein and gene therapies. Current available preclinical studies have shown efficacy of protein-encoding cmRNA in the regeneration or repair of craniomaxillofacial and long bone defects, tendon defects or injuries, OA and IVDD, and skeletal muscle injuries using various functional target genes, but no clinical trial has been initiated. However, comprehensive studies addressing the choice of growth factors, doses, and delivery strategies are still needed. Additionally, further information regarding long-term safety, efficacy, and translational feasibility are necessary to fully understand the therapeutic potential of mRNA therapy for musculoskeletal regeneration before clinical translation of this research.

## Figures and Tables

**Table 1:** Summary of protein-encoding mRNA for musculo-skeletal tissue repair.

Target Tissue	Encoded Protein	Delivery Modality /Platform	Scaffold Materials	Study Type	Key Finding	References
Critical-sized calvarial bone defect	BMP-2, FGF-2, PDGF-BB cmRNA (U→s2U (25%), C→m5C (25%) and U→Ψ (100%), C→m5C (100%))	PEI cmRNA polyplexes	Collagen	In vivo/in vitro	BMP-2 cmRNA advantageous over BMP-2 pDNA Co-delivery of FGF-2 and PDGF-BB mRNAs suppressed effects of BMP-2 cmRNA	[19]
	BMP-2 (cmRNA, U→m1Ψ (100%))	LNPs	Silk fibroin (SF)/gelatin silk fibroin (G)	In vivo/in vitro	BMP-2 cmRNA advantageous over pDNA BMP-2	[14]
	BMP-9 cmRNA (U→Ψ (100%), C→m5C (100%))	PEI cmRNA nanoplexes	Cross-linked collagen membranes	In vivo/in vitro	BMP-9 cmRNA enchances bone regeneration	[20]
	BMP-9, BMP-2 cmRNA (U→Ψ (100%), C→m5C (100%))	PEI cmRNA polyplexes	Collagen	In vivo/in vitro	BMP-9 may be more effective in bone regeneration than BMP-2	[21]
	VEGF, BMP-2 cmRNA (U→m1\Ψ (100%))	Transfected BMSCs	Collagen	In vivo/in vitro	VEGF and BMP-2 cmRNA transfected rBMSCs synergistically enhanced bone defect repair	[22]
	BMP-2, TGFβ-3 mRNA	PEG-PAsp(DET) block copolymer nanomicelles	Gelatin Sponge	In vivo/in vitro	Potential synergistic effects between BMP-2 and TGFβ-3 in enhancing bone repair	[23]
	Runx2, VEGF mRNA	PEG-PAsp(DET) nanomicelles	N/A	In vivo/in vitro	Runx2 and VEGF individually enhanced bone regeneration and synergistic effects were observed with their co-administration	[26]
	BMP-2 mRNA	Exosomes	GelMA Hydrogel	In vivo/in vitro	BMP-2 enriched exosomes loaded on a hydrogel scaffold promotes osteogenesis of critical bone defects.	[27]
Non-critical-size long bone defect	BMP-2 cmRNA (U→s2U (25%), C→m5C (25%))	C12-EPE/hNP-2 cmRNA lipoids	Fibrin gel	In vivo/in vitro	hBMP-2 cmRNA accelerated bone healing	[30]
	BMP-2 cmRNA (U→m1Ψ (100%))	PEI cmRNA polyplexes	Vacuum dried collagen sponges	In vivo/in vitro	hBMP-2 delivery by TAMs lead to prolonged protein delivery and accelerated bone healing	[31]
	β-catenin^GOF^ cmRNA (U→m1Ψ (100%))	SM-102 lipid nanoparticles	N/A	In vivo/in vitro	SM-102 β-catenin^GOF^ injection increased bone formation in murine tibia fracture model	[32]
Critical-size long bone defect	BMP-2 cmRNA (U→5IU (35%) C→5IC (7.5%))	cmRNA lipoplexes	Collagen sponge	In vivo	BMP-2 cmRNA can regenerate bone with no off-target effects or ectopic callus formation	[34]
	BMP-2, VEGF-A mRNA (native)	Therapeutic small extracellular vesicles (t-sEVs)	Injectable PEGylated poly (glycerol sebacate) acrylate (PEGS-A) hdyrogel	In vitro/in vivo	Localized release of BMP-2 mRNA by t-sEVs leads to highly efficient bone regeneration with minimal off-target effects	[35]
Traumatic osteoarthritis	Runx1 mRNA	PEG-PAsp(TET), PEG-PAsp(DET)	N/A	In vivo	Runx1 suppresses OA progression more effectively at early stages of OA by acting on remaining chondrocytes.	[42]
	Runx1 mRNA	PEG-PAsp(DET)	N/A	In vivo	Raman spectroscopic analysis confirmed Runx1 mediated cartilage regeneration through activation of remaining chondrocytes.	[43]
	IGF-1 cmRNA (U→m1Ψ (100%))	Transfected ADSCs	N/A	In vivo/in vitro	Transfected ADSCs ameliorated OA progression in DMM OA model	[44]
	Circular FGF-18 mRNA	Biodegradable and ionizable glycerolipid, TG6A, with branched tails and five ester bonds LNP-mRNA transfected MSCs	N/A	In vivo/in vitro	Enhanced MSCs cell proliferation and chondrogenic differentiation in vitro and promote cartilage repair in rat DMM induced OA.	[46]
	rhFGF-18 cmRNA (unspecified modifications)	WG-PL14 LNP	N/A	In vivo/in vitro	LNP-rhFGF18 mRNA treatment in murine OA model ameliorated OA progression at low dose (2 µg)	[47]
	FGF-18 cmRNA (U→m1Ψ (100%))	LNPs made with 4 lipids including SM-102.	N/A	In vivo/in vitro	LNP-FGF-18 cmRNA protected chondrocytes from degeneration and senescence, improving OA symptoms via the FOXO3a-autophagy pathway	[37]
Osteochondral defect	TGFβ-1 cmRNA (U→Ψ (100%), C→m5C (100%))	Mineral coated microparticles (MCMs) and fluoride MCMs (FMCMs)	Transfected bone marrow aspirate concentrate (BMAC) + peripheral blood clot	In vivo/in vitro	Reduced fibrocartilage formation, positively influencing the regenerative potential of autologous BMAC	[49]
Intervetebral disc	Runx1 mRNA	PEG-PAsp(Det) nanomicelles	N/A	In vivo	Maintained disc height and hydration content and prevented fibrous tissue formation	[50,51]
	IGF-1 mRNA construct (NTF3 signal peptide, pro-human IGF-1 domain, full-length IGF-1 mRNA coding sequence)	Naked	N/A	In vivo, in vitro	IGF-1 mRNA construct (Cpd.3) in simple buffer ameliorated intervertebral disc degeneration	[52]
Achilles tendon defect	BMP-7 cmRNA (Unspecified modification)	Naked	N/A	In vivo, ex vivo	cmRNA treatment of surgically repaired defect resulted in significantly high BMP-7 expression at day 2 and decreased collagen III expression in regenerating tissues at day 7	[54]
	bFGF cmRNA (U→s2U (25%) C→m5C (25%))	Naked	N/A	In vivo	cmRNA treatment of non-surgically repaired tendon resulted in increased tendon stiffness with no side effects	[55]
Patella tendon defect	PDGF-BB and IL1RA cmRNAs (U→5IU (35%) C→5IC (7.5%))	poly(amidoamine)-based polymers (ps-PAAQ) as NP carriers	N/A	in vivo, in vitro	Combined application IL1RA and PDGF-BB cmRNAs reduced inflammation and fibrotic markers and ehanced repair of tissue structures	[57]
Tendinopathy	cmRNA^Luc^ (unspecified modifications)	Naked	N/A	in vivo	Target gene expression did not increase over time in intact tendon, while dose dependent expression was found in the injured tendons	[54]
	TdTomato cmRNA (unspecified modifications)	Cationic, hyperbranched poly(amindoamine)-based nanoparticles	N/A	In vitro	Moderate amounts of NPs enhanced transfection efficiency in rTDSPCs while higher doses caused cytotoxicity	[56]
	IL1RA mRNA	SM-102 lipid nanoparticles	N/A	In vitro, in vivo	Reduced inflammatory markers, reversed matrix degradation, and promoted functional recovery	[58]
Skeletal Muscle myotoxic model and muscle punch model	IGF-1 mRNA construct (BDNF signal peptide, pro-domain of hIGF-1 and full length IGF-1mRNA coding sequence)	Naked	N/A	In vitro, in vivo	IGF-1 cmRNA construct (Cpd.2) was more potent than natural IGF-1 mRNA in muscle healing and regeneration in two mouse muscle injury models	[52]

Only mRNAs with modified nucleotides were classified as cmRNAs. mRNAs include synthesized molecules that contain unaltered target-protein sequences often with an added 5′ cap and 3′ poly-A tail. mRNA (native) indicates non-synthesized mRNAs. Denotation: **U→s2U** = Uridine → 2-thiouridine. **C→m5C** = Cytidine → 5-methylcytidine. **U→m1Ψ** = Uridine → N1-methylpseudouridine. **U→5IU**= Uridine → 5-iodouridine. **C→5IC** = Cytidine → 5-iodocytidine. N/A, not applicable.

## Data Availability

No new data were created or analyzed in this study. Data sharing is not applicable to this article.
